# Blood–Brain Barrier Leakage Is Increased in Parkinson’s Disease

**DOI:** 10.3389/fphys.2020.593026

**Published:** 2020-12-22

**Authors:** Sarah Al-Bachari, Josephine H. Naish, Geoff J. M. Parker, Hedley C. A. Emsley, Laura M. Parkes

**Affiliations:** ^1^Lancaster Medical School, Faculty of Health and Medicine, Lancaster University, Lancaster, United Kingdom; ^2^Department of Neurology, Lancashire Teaching Hospitals NHS Foundation Trust, Preston, United Kingdom; ^3^Division of Neuroscience and Experimental Psychology, Faculty of Biology, Medicine and Health, The University of Manchester, Manchester, United Kingdom; ^4^Division of Cardiovascular sciences, Faculty of Biology, Medicine and Health, The University of Manchester, Manchester, United Kingdom; ^5^Bioxydyn Limited, Manchester, United Kingdom; ^6^Centre for Medical Image Computing, Department of Computer Science and Department of Neuroinflammation, University College London, London, United Kingdom; ^7^Geoffrey Jefferson Brain Research Centre, Manchester Academic Health Science Centre, Manchester, United Kingdom

**Keywords:** blood–brain barrier, cerebrovascular disease, dynamic contrast enhanced MRI, Parkinson’s disease, neurovascular unit

## Abstract

**Background:**

Blood–brain barrier (BBB) disruption has been noted in animal models of Parkinson’s disease (PD) and forms the basis of the vascular hypothesis of neurodegeneration, yet clinical studies are lacking.

**Objective:**

To determine alterations in BBB integrity in PD, with comparison to cerebrovascular disease.

**Methods:**

Dynamic contrast enhanced magnetic resonance images were collected from 49 PD patients, 15 control subjects with cerebrovascular disease [control positive (CP)] and 31 healthy control subjects [control negative (CN)], with all groups matched for age. Quantitative maps of the contrast agent transfer coefficient across the BBB (*K*^trans^) and plasma volume (v_*p*_) were produced using Patlak analysis. Differences in *K*^trans^ and v_*p*_ were assessed with voxel-based analysis as well as in regions associated with PD pathophysiology. In addition, the volume of white matter lesions (WMLs) was obtained from T_2_-weighted fluid attenuation inversion recovery (FLAIR) images.

**Results:**

Higher *K*^trans^, reflecting higher BBB leakage, was found in the PD group than in the CN group using voxel-based analysis; differences were most prominent in the posterior white matter regions. Region of interest analysis confirmed *K*^trans^ to be significantly higher in PD than in CN, predominantly driven by differences in the substantia nigra, normal-appearing white matter, WML and the posterior cortex. WML volume was significantly higher in PD compared to CN. *K*^trans^ values and WML volume were similar in PD and CP, suggesting a similar burden of cerebrovascular disease despite lower cardiovascular risk factors.

**Conclusion:**

These results show BBB disruption in PD.

## Introduction

The blood–brain barrier (BBB) consists of highly specialised, metabolically active cells forming a selectively permeable, highly resistant barrier to diffusion of blood products (Pardridge, 2005). It is closely coupled with glial cells (i.e., pericytes, microglia, oligodendroglia, and astrocyte end-feet), all in close proximity to a neuron; collectively termed the neurovascular unit (Lo and Rosenberg, 2009; Alvarez et al., 2013). Normal functioning of the neurovascular unit ensures healthy function of the BBB and adequate cerebral blood flow, it also maintains the neuronal “milieu” which is required for proper functioning of neuronal circuits and ensures the metabolic needs of the neurons are met (Zlokovic, 2008, 2011). In the neurovascular unit, BBB permeability and cerebral blood flow are mainly controlled by endothelial cells, smooth muscle cells and pericytes; damage to which have been associated with accumulation of neurotoxins and hypoxia leading to neuronal injury and loss (Bell et al., 2010; Montagne et al., 2018).

Neurodegeneration is now understood to be the consequence of multiple factors acting and interacting over time to lead to neuronal dysfunction and death (Carvey et al., 2006; Collins et al., 2012; Sweeney et al., 2019). Neurovascular unit dysfunction, unsurprisingly, contributes to neuronal dysfunction and death; this forms the basis of the “vascular model of neurodegeneration” (Grammas et al., 2011; Zlokovic, 2011; Andreone et al., 2015; Nelson et al., 2015; Zhao et al., 2015; Hachinski et al., 2019). The two pillars of this model are hypoperfusion and BBB disruption, both contributing to the vicious circle of neuronal loss. Studies particularly in the preclinical setting, suggest microvascular pathology and hypoperfusion occurs in the context of neurodegenerative diseases (Brown and Thore, 2011; Zlokovic, 2011; Di Marco et al., 2015; Iadecola, 2017; Kisler et al., 2017). In addition studies in Parkinson’s disease (PD) have revealed vascular remodelling, altered vasculature and abnormal angiogenesis (Faucheux et al., 1999; Farkas et al., 2000; Wada et al., 2006; Chao et al., 2009; Patel et al., 2011; Guan et al., 2013; Janelidze et al., 2015).

Understanding of the pathogenesis of PD centres around the selective and progressive loss of dopaminergic neurons in the substantia nigra pars compacta (SNpc) and its connections with other basal ganglia structures. BBB disruption contributing to neurodegeneration in the SNpc has been reported in PD in animal studies (Barcia et al., 2005; Rite et al., 2007; Chao et al., 2009). In humans, a relatively small positron emission tomography (PET) study in PD patients revealed dysfunction of the BBB transporter system (Kortekaas et al., 2005). A histological study revealed significantly increased permeability of the BBB in the post commissural putamen of PD patients (Gray and Woulfe, 2015). Thus the areas implicated in PD pathology have been shown to demonstrate BBB disruption, yet studies remain few and predominantly in animal models.

Many studies describe hypoperfusion in the posterior cortices in PD, in particular in the posterior parieto-occipital cortex, pre-cuneus and cuneus and temporal regions with variable patterns in the frontal lobe (Ma et al., 2010; Kamagata et al., 2011; Melzer et al., 2011; Borghammer, 2012; Fernandez-Seara et al., 2012). The extent to which BBB disruption impacts perfusion and vice versa (hypoperfusion influencing BBB disruption) is poorly understood. However, both occur at the microvascular level and may be linked. If this is the case, this then suggests that alterations in BBB may also be expected in these posterior regions as well as in regions implicated in PD pathology such as the basal ganglia, where neuronal loss and loss of nigrostriatal projections occurs.

Advances in neuroimaging techniques, in particular quantitative MRI techniques such as arterial spin labelling and dynamic contrast enhanced magnetic resonance imaging (DCE-MRI), have paved the way for studies of the microcirculation in the clinical setting, with DCE-MRI specifically probing BBB integrity (Tofts and Kermode, 1991). Previously applied to measure BBB disruption in tumours, multiple sclerosis and acute ischaemic stroke, recent applications have used this technique to probe more subtle and chronic BBB disruption. Studies include small vessel disease (Wardlaw et al., 2008), Alzheimer’s disease (Starr et al., 2009), mild cognitive impairment and normal ageing (Montagne et al., 2015), vascular cognitive impairment (Taheri et al., 2011) and diabetes (Starr et al., 2003); its value in these settings has been systematically reviewed (Heye et al., 2014). To our knowledge there is no published work on DCE-MRI measures in PD.

We used DCE-MRI to investigate regional alterations in BBB permeability in the context of PD. PD was compared with a control group with known cerebrovascular disease [control positive (CP)] and a control group without known cerebrovascular disease or PD [control negative (CN)]. Our aim was to investigate whether potential changes are simply attributable to co-existing cerebrovascular disease in an ageing population or if a pattern of BBB alterations specific to PD is revealed. Inclusion of the CP group allows us to do this by comparing the pattern of BBB disruption in the PD and CP groups with reference to the burden of cerebrovascular disease in each group, defined by white matter lesion (WML) volume as an accepted surrogate marker of small vessel disease. We hypothesised that BBB disruption in PD would occur in the basal ganglia structures due to the pathophysiology of PD being centred around selective and progressive loss of dopaminergic neurons in the SNpc and nigrostriatal pathways. Therefore, based on the vascular hypothesis of neurodegeneration, these areas should display BBB disruption. We also expected BBB disruption to occur in posterior and frontal cortices given that hypoperfusion, which potentially impacts BBB function, has been noted in these regions in PD. Finally, as BBB alterations in cerebrovascular disease have been found within WML and in the normal appearing white matter (NAWM) (Wardlaw et al., 2017) we also considered alterations in these regions. Hence we investigated BBB changes in basal ganglia, posterior and frontal cortex regions, NAWM and WML, along with a more exploratory voxel-wise analysis across the entire brain.

## Materials and Methods

### Approvals, Recruitment, Eligibility and Consent

Relevant approvals were obtained including National Health Service (NHS) ethical approval (North West – Preston Research Ethics Committee), research governance and local university approvals. PD patients were recruited from Lancashire Teaching Hospitals NHS Foundation Trust and Salford Royal NHS Foundation Trust. Eligibility criteria for PD participants were a clinical diagnosis of PD fulfilling United Kingdom PD society brain bank criteria^1^ without known clinical cerebrovascular disease (no history of transient ischaemic attack or stroke) or dementia (Emre et al., 2007). Participants with cerebrovascular disease were recruited from patients attending Lancashire Teaching Hospitals with a clinical diagnosis of stroke or transient ischaemic attack within the previous 2 years (and at least 3 months prior to participation) supported by relevant brain imaging (CP). Controls without a history of either PD or clinical cerebrovascular disease were also recruited from the local community (CN). All groups were matched for age. All participants were required to provide written informed consent and had capacity to do so.

### Clinical Assessments

Parkinson’s disease assessment included the Unified Parkinson’s Disease Rating Scale (UPDRS)^2^ during the scan visit. Disease severity was measured using the Hoehn and Yahr rating scale (Hoehn and Yahr, 1967). No alterations were made to the participants’ medications for the study protocol. Routine clinical baseline data were also recorded and the levodopa equivalent daily dose (LEDD) calculated (Tomlinson et al., 2010). A battery of clinical scales was also administered, including the Montreal Cognitive Assessment (MoCA)^3^ to measure cognition. Demographics and clinical data were compared between PD and control participants using unpaired Student’s *t*-test for continuous variables or Fisher’s exact test for categorical variables with *p* value set at <0.05.

### MRI Protocol

Participants were scanned on one of two systems running the same software version: a 3.0 T Philips Achieva scanner with an eight channel head coil at Salford Royal Hospital or a separate 3.0 T Philips Achieva scanner with a 32 channel head coil at the Manchester Clinical Research Facility. Involuntary movements in participants were minimised using padding within the head coil.

Both scanners ran an identical MRI protocol. A DCE-MRI dynamic series of 160 3D T_1_-weighted images [T_1_ Fast Field Echo (T_1_-FFE)] were acquired with a temporal resolution of 7.6 s, spatial resolution of 1.5 × 1.5 × 4 mm, and total duration of approximately 20 min. On the 8th dynamic, a gadolinium-based contrast agent (Dotarem) bolus was administered using a power injector. The volume administered was proportional to the weight of the subject with a dose of 0.1 mmol/kg.

Prior to the dynamic scan, a series of additional 3D T_1_-FFE images were acquired at three flip angles (2, 5, and 10 degrees) in order to calculate a pre-contrast T_1_ map using the variable flip angle method. A B_1_ map was also collected in order to correct for B_1_ field inhomogeneities.

In addition, a 1 mm isotropic 3D T_1_-weighted image and a T_2_-weighted fluid attenuation inversion recovery (FLAIR) image were acquired. Please see supplementary material for full details of acquisition parameters.

### MRI Analysis

#### White Matter Lesion Volume Estimation

White matter lesion volume was calculated as an established marker of small vessel disease (Wardlaw et al., 2013). WML volume was estimated using the lesion segmentation toolbox (Schmidt et al., 2012) in SPM8 using both T_2_-weighted FLAIR images and T_1_-weighted images as inputs. A threshold of 0.3 was chosen as it gave the most accurate estimates in a sub-study comparing WML volume estimates from the lesion segmentation toolbox with those from semi-automated lesion-growing methods on a subset of the data (*n* = 51, including representation from all groups, unpublished)^4^. WML volumes were positively skewed and were therefore cube-root transformed as is commonly done (Stefaniak et al., 2018) before group comparisons using un-paired *t*-tests.

#### DCE Analysis

The dynamic series of 160 images were first corrected for motion using the “realignment” option in SPM12^5^, which aligned all DCE-MRI images to the first image in the time-series. A vascular input function was derived from the sagittal sinus (Lavini and Verhoeff, 2010), which was delineated using MRIcro on the final image of the motion-corrected dynamic time series. Regions of approximately 50 voxels were selected. A voxel-by-voxel fit of the dynamic data for both the contrast agent transfer coefficient (*K*^trans^) and plasma volume (v_*p*_) was performed using the uptake or “Patlak” model assuming unidirectional transport of the tracer from the blood plasma to the extravascular, extracellular space. Further details regarding DCE-MRI analysis can be found in Supplementary Material.

Mean images of *K*^trans^ and v_*p*_ in each of the three groups were created following spatial smoothing using a 3D 3 mm full-width-half-maximum kernel in and visually inspected for differences. Voxel-wise analysis was performed using the SPM12 PET toolbox to determine regional differences in *K*^trans^ and v_*p*_ between the groups. *K*^trans^ and v_*p*_ maps were co-registered to the high resolution 3D T_1_-weighted image and then normalised to Montreal Neurological Institute (MNI) space. The normalised *K*^trans^ and v_*p*_ maps were spatially smoothed using an 8 mm full-width-half-maximum kernel. Voxel-wise comparisons of *K*^trans^ and v_*p*_ between the groups were performed, without intensity normalisation, using a two-sample unpaired *t*-test (unequal variances). Group comparisons were performed between: CN and PD, CP and PD and CP and CN. Regions were considered to show significant group differences at a voxel-level threshold of *p* < 0.001 uncorrected, and a minimum cluster size of 50 voxels, masked to the intra-cranial volume. Further analysis using family wise error (FWE) correction for multiple comparisons at the cluster level was performed. The MNI coordinates were used to identify regions showing group differences using xjview V 8.14^6^.

**FIGURE 1:**
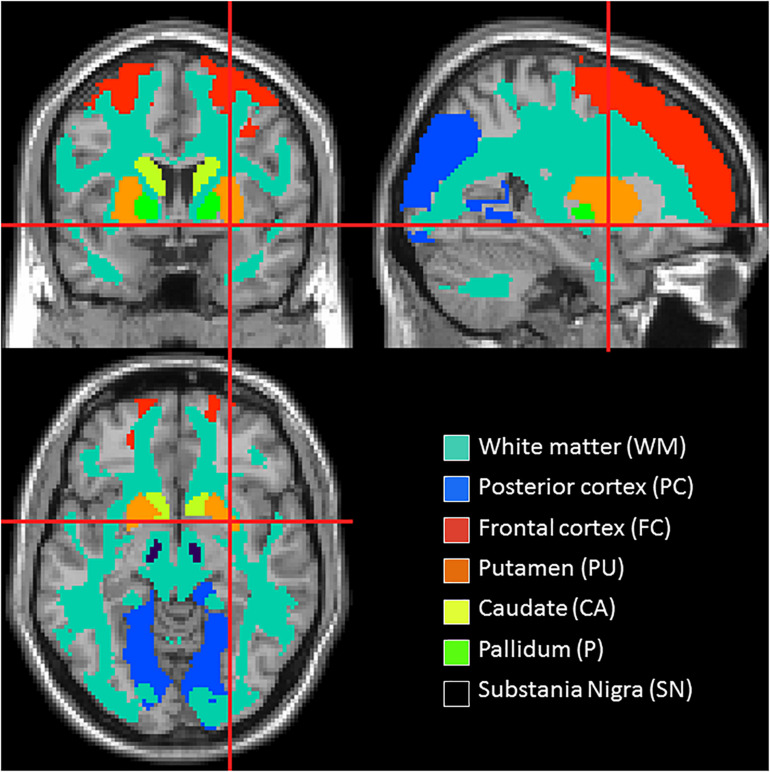
Location of the regions of interest.

Group differences in *K*^trans^ and v_*p*_ were also assessed in region of interest (ROI) including the basal ganglia, frontal and posterior cortices, WML and NAWM. WML regions were obtained using the co-registered binary lesion masks from the lesion segmentation (see section “White Matter Lesion Volume Estimation”) and care was taken to remove regions of WML from all other ROIs. The caudate (CA), putamen (PU), and pallidum (P) regions were obtained from MNI atlases (Tzourio-Mazoyer et al., 2002; Prodoehl et al., 2008). The substantia nigra (SN) region was manually drawn on the T_2_-weighted template image from SPM by an experienced researcher. Frontal and selected posterior cortical regions were also defined (in keeping with regions of hypoperfusion in other studies) (Melzer et al., 2011; Fernandez-Seara et al., 2012; Gray and Woulfe, 2015; Al-Bachari et al., 2017) using a combination of regions from the automatic anatomical labelling atlas (Tzourio-Mazoyer et al., 2002). The frontal region consisted of superior and middle frontal gyri and the posterior region of pre-cuneus, cuneus, lingual, superior, and middle occipital gyri. Finally, NAWM was also selected, defined using the mask from the segmentation of the co-registered T_1_-weighted image, in order to determine the significance of any diffuse differences between the groups. Figure 1 depicts the location of these ROIs. Mean *K*^trans^ and v_*p*_ values were extracted from each of these regions for each subject. Repeated measures analysis of variance (ANOVA) was performed with factors “group” and “region” to determine any significant difference in *K*^trans^ and v_*p*_ between the groups with “subject” included as a random effect. A second ANOVA was performed with the addition of age, gender and the cube-root of WML volume as covariates to determine if these factors could explain any variance in *K*^trans^ and v_*p*_. Where significant group differences were found, *post hoc t*-tests were performed with Bonferroni correction where stated. Statistical analyses were conducted using R-Studio Version 1.3.959^7^.

#### Correlation With Cognitive and Clinical Parameters

Any association between the DCE-MRI parameters and cognitive deficit, medication and disease severity within the PD group was evaluated with a linear mixed effects model and ANOVA. Region (as a factor), MoCA score, LEDD dose and UPDRS score (as continuous variables) and their interaction with region were modelled as fixed effects, and subject was set as a random effect. Where significant interactions with region were found, the fixed effects *t*-tests and corresponding *p* values for each region were considered, calculated using Satterthwaite’s approximation in the lmerTest package (Kuznetsova et al., 2017) in R-studio.

## Results

Fifty-one PD patients were recruited, 17 CP subjects with cerebrovascular disease (13 with ischaemic stroke, 4 with single or multiple transient ischaemic attacks; mean time since symptom onset and where applicable most recent known transient ischaemic attack = 1.1 ± 0.7 years) and 34 CN subjects. Twenty-eight participants were scanned at Salford Royal Hospital and 74 participants at the Manchester Clinical Research Facility (37 PD, 20 CN, and 17 CP).

Data from seven participants could not be analysed due to (i) participants not tolerating the complete scan procedure (*n* = 2) (ii) failure of the contrast agent injection (*n* = 3), resulting in either absent, very low or very distorted vascular input function and (iii) non-physiological values of plasma volume (*n* = 2); leaving data from 95 participants (49 PD, 31 CN, 15 CP). Summary demographic information from these patients is given in Table 1, along with the WML volume measurements. There were no significant differences in age between the groups. As expected the CP group had more cerebrovascular risk factors than either the PD or CN groups but there was no difference in risk factors between the PD and CN groups. WML volume was significantly higher in the PD group than the CN group, suggesting that, although vascular risk factors are similar, there was increased microvascular pathology in the PD group. WML volume was also higher in the CP group that the CN group, as expected, but not significantly different from the PD group. The PD group had significantly lower MoCA score compared to the CN group, but was not significantly different from the CP group. It is noted that there are significant gender differences between the PD and CN groups, which is addressed directly in a sub-analysis (see Supplementary Materials).

**TABLE 1:** Demographics and clinical and radiological characteristics of the study group.

	CN (*n* = 31)	CP (*n* = 15)	PD (*n* = 49)	*p* value PD vs. CN	*p* value PD vs. CP	*p* value CP vs. CN
*n* (F:M)	16:15	4:11	12:37	0.01	0.25	0.07
Age (years): mean (range)	66.4 (52–81)	69.1 (53–84)	68.9 (52–85)	0.23	0.84	0.26
No. of cardiovascular risk factors: mean (SD)	1.52 (1.12)	2.93 (1.16)	1.72 (1.52)	0.55	0.002	<0.0001
Cardiovascular Risk Factors (% of group): Hypertension Diabetes mellitus FH of CVD Smoker Hypercholesterolaemia Ischaemic heart disease Atrial fibrillation	29.0 6.5 45.2 29.0 45.2 6.5 0	73.3 13.3 46.7 66.7 68.9 13.3 20.0	26.5 6.1 22.4 28.6 22.4 12.2 2.0	0.13 0.36 0.10 0.15 0.08 0.13 0.61	0.02 0.43 0.15 0.03 0.004 0.31 0.04	0.005 0.46 0.25 0.01 0.05 0.30 0.03
Disease Duration (years): mean (SD)	N/A	1.1 (0.77)	7.2 (4.45)	N/A	N/A	N/A
Hoehn and Yahr Score: mean (SD)	N/A	N/A	2.60 (0.09)	N/A	N/A	N/A
UPDRS Score: mean (SD)	N/A	N/A	29.2 (12.7)	N/A	N/A	N/A
LEDD (mg): mean (SD)	N/A	N/A	583.5 (399.6)	N/A	N/A	N/A
MoCA Score: mean (SD)	27.9 (2.3)	26.1 (2.9)	25.2 (3.9)	0.0004	0.39	0.04
Cube-root of WML volume (mm): mean (SD)	1.26 (0.83)	2.11 (0.72)	1.80 (0.95)	0.008	0.19	0.001

*AF, atrial fibrillation; CN, control negative; CP, control positive; CV, cardiovascular; DM, diabetes mellitus; FH of CVD, family history of cardiovascular disease; Hyperchol., hypercholesterolaemia; IHD, ischaemic heart disease; LEDD, levodopa equivalent daily dose; MoCA, Montreal Cognitive Assessment; RF, risk factors; PD, Parkinson’s disease; TD, tremor dominant; UPDRS 111, unified Parkinson’s disease rating scale motor score; WML, white matter lesion.*

### Voxel-Wise Analysis

**FIGURE 2:**
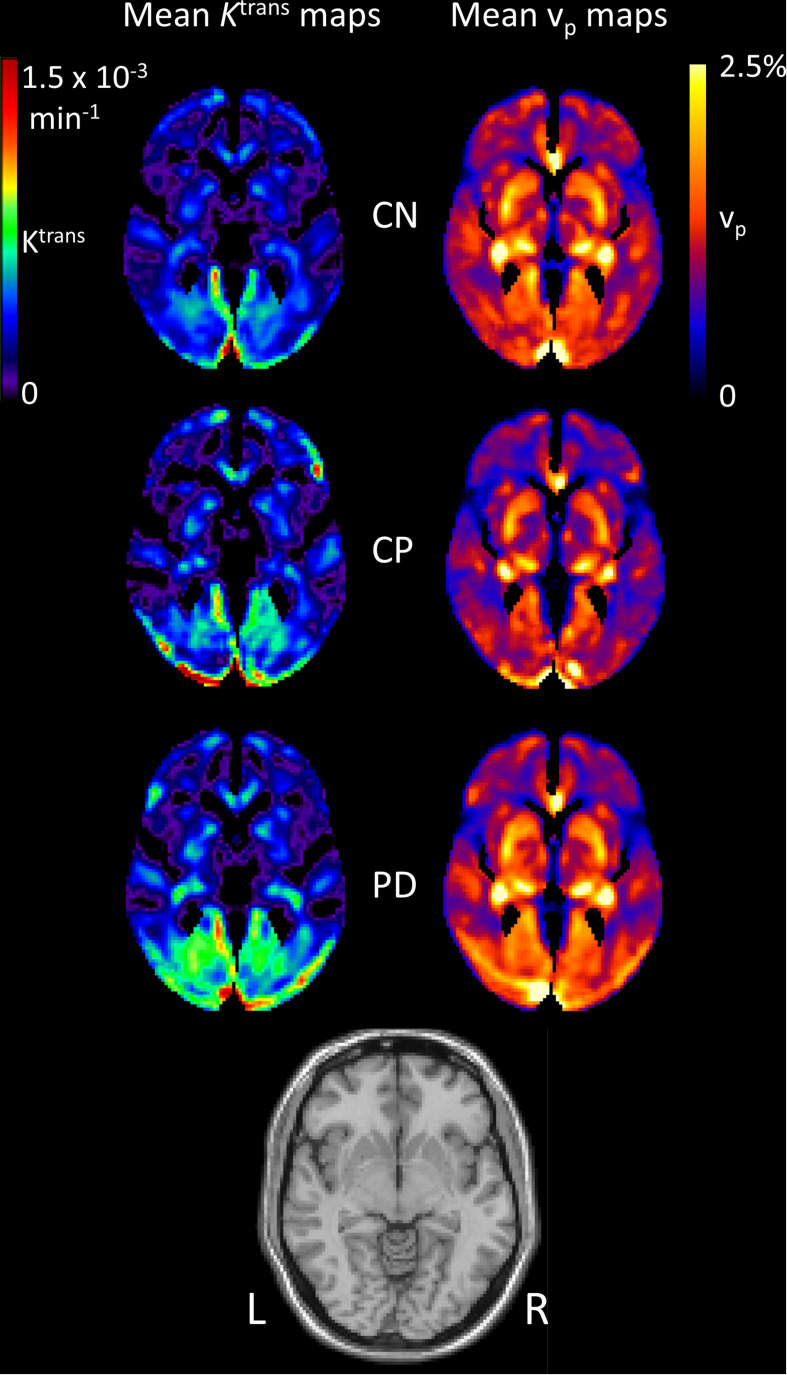
Mean images of *K*^trans^ and v_*p*_ for each group. Images of the mean contrast agent transfer coefficient *K*^trans^ and the plasma volume v_*p*_ for each group. Individual images were first normalised to MNI space before averaging. A T_1_-weighted image is shown for reference.

Figure 2 shows mean images of *K*^trans^ and v_*p*_ in the three groups. It can be seen that *K*^trans^ is generally higher in PD than in the control groups. The v_*p*_ maps look similar between the PD and CN group, but the CP group has noticeably lower v_*p*_.

**FIGURE 3:**
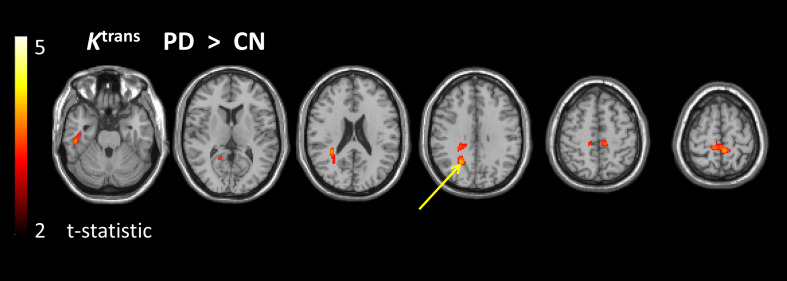
Regions of higher *K*^trans^ in the PD group compared to the CN group. t-statistic map overlaid on structural image showing the regions of significantly higher *K*^trans^ in the PD group than in the CN group. Map is thresholded with voxel-level *p* < 0.001 (uncorrected) and minimum cluster size of 50 voxels. The arrow indicates the cluster that survives cluster-level family wise error correction for multiple comparisons (*p* < 0.05).

The voxel-wise comparisons revealed significantly higher *K*^trans^ in the PD group than in the CN group (Figure 3 and Supplementary Table 1) in regions including white matter regions of the pre-cuneus bilaterally. Only the largest region in the right pre-cuneus survived cluster-level FWE correction. There were no regions of significantly lower *K*^trans^ in PD than in CN. *K*^trans^ was also significantly higher in the PD group than in the CP group in one region of white matter in the right temporal lobe (Supplementary Table 2). Significantly higher *K*^trans^ was also seen in the CP group than in the CN group (Supplementary Table 3), in the mid cingulum and R cerebellum. Aside from for the PD vs. CN comparison, none of these regions survived the cluster-level FWE correction.

Control positive showed regions of significantly lower v_*p*_ than CN (Supplementary Table 4) and PD (Supplementary Table 5) in white matter regions of the left and right temporal lobes. No significant voxel-wise differences in v_*p*_ were seen for PD vs. CN.

### ROI Analysis

**FIGURE 4:**
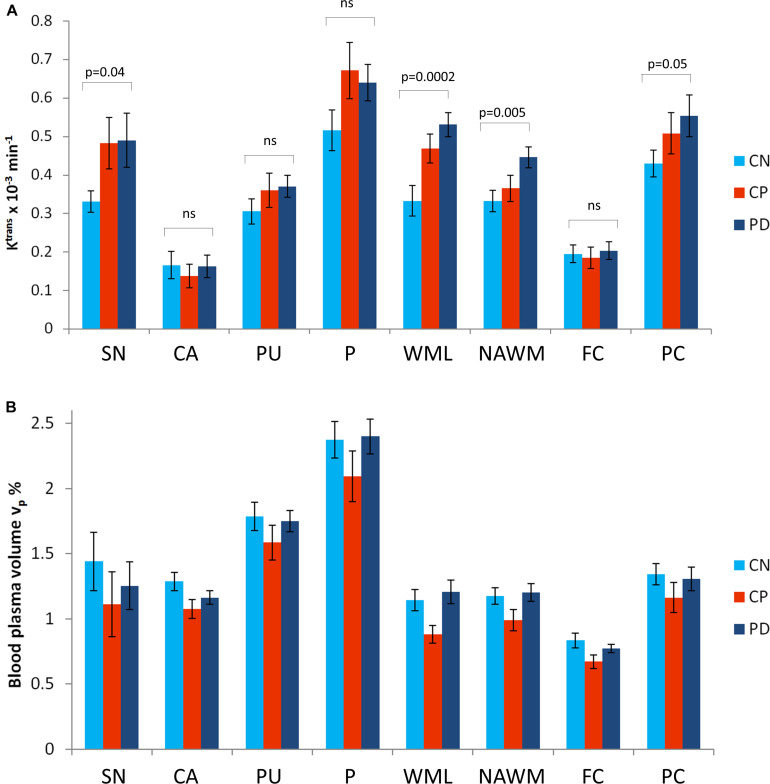
Mean values for *K*^trans^ and v_*p*_ in regions of interest for each group. Mean values are given for **(A)** the contrast agent transfer coefficient *K*^trans^ and **(B)** the plasma volume v_*p*_. Error bars show the standard error in the mean. The significance of *post hoc t*-tests (uncorrected) between *K*^trans^ in the PD and CN group are shown. SN, substantia nigra; CA, caudate; PU, putamen; P, pallidum; WMLs, white matter lesions; NAWM, normal-appearing white matter; FC, frontal cortex; PC, posterior cortices.

Figure 4 shows group mean regional values for *K*^trans^ and v_*p*_. There was a significant effect of group (*F* = 3.3, *p* = 0.04) and region (*F* = 54.1, *p* < 0.0001) on *K*^trans^ with *post hoc* tests showing *K*^trans^ to be significantly higher in PD than in CN (*p* = 0.03, Bonferroni corrected) and no significant differences between the other two pairwise comparisons. The NAWM, posterior cortex and SN show elevated *K*^trans^ in PD compared to CN when considering differences on a region-by-region basis (*p* < 0.05, uncorrected). *K*^trans^ is also higher *within* the WML in PD in comparison to CN. A second ANOVA with WML volume, age and gender included as covariates showed a similar effect of group (*F* = 3.9, *p* = 0.02) and region (*F* = 54.1, *p* < 0.0001) and no significant effect of WML volume (*F* = 1.0 *p* = 0.3), age (*F* = 1.1, *p* = 0.3) or gender (*F* = 0.1, *p* = 0.8). *Post hoc* tests again showed *K*^trans^ to be significantly higher in PD than in CN (*p* = 0.02, Bonferroni corrected).

There was a significant effect of region (*F* = 90.0, *p* < 0.0001) but not group (*F* = 1.1, *p* = 0.3) on v_*p*_. The second ANOVA with WML volume, age and gender included as covariates showed a similar result with an impact of region (*F* = 90.0, *p* < 0.0001) but not group (*F* = 1.1, *p* = 0.3) on v_*p*_ and no significant effect of WML volume (*F* = 0.1. *p* = 0.8), age (*F* = 2.2, *p* = 0.1) or gender (*F* = 0.2, *p* = 0.6).

To check that differences were not driven by the differences in gender-matching or by the use of two scanners, the regional analysis was repeated with gender-matched groups and with data from only one scanner. Broadly the same regional and group effects were seen for both *K*^trans^ and v_*p*_ (see supplementary materials).

### Correlation With Cognitive and Clinical Parameters

Table 2 summaries the ANOVA findings evaluating the impact of cognitive deficit (MoCA score), medication (LEDD dose) and disease severity (UPDRS score) on the DCE-MRI parameters within the PD group. There are no significant associations between these parameters and *K*^trans^. In particular, LEDD dose was not associated with *K*^trans^ suggesting that the increased BBB leakage seen in the PD group is not a consequence of medication. A significant effect of LEDD dose on v_*p*_ was found with higher LEDD dose associated with higher v_*p*_.

**TABLE 2:** Analysis of variance for the impact of cognitive deficit (MoCA score), medication (LEDD dose) and disease severity (UPDRS score) on regional DCE-MRI parameters within the PD group.

Factor		*K*^trans^ as dependent variable		*V*_*p*_ as dependent variable	
			
	Deg. freedom	*F* value	*p* value	*F* value	*p* value
Region	6	0.3	1.0	3.5	0.002
MoCA	1	3.8	0.06	0.4	0.52
LEDD	1	0.4	0.5	6.1	0.02
UPDRS	1	0.5	0.5	0.02	0.9
Region: MoCA	6	0.1	1.0	1.7	0.1
Region: LEDD	6	0.5	0.8	1.0	0.4
Region: UPDRS	6	0.4	0.9	0.8	0.5

## Discussion

The aim of this study was to determine alterations in BBB permeability in PD, by comparison with controls, and to investigate whether potential changes are simply attributable to co-existing cerebrovascular disease in an ageing population or if a pattern of BBB alteration specific to PD is revealed. The results show higher *K*^trans^, reflecting higher BBB leakage, in PD than in CN (Figures 2, 3 and Supplementary Table 1), with a somewhat different spatial pattern to the differences seen between individuals with known cerebrovascular disease (CP) and CN (Supplementary Table 3). Direct comparison of PD and CP shows higher *K*^trans^ for PD in the white matter of the right temporal lobe (Supplementary Table 2). Blood plasma volume, v_*p*_, is similar in PD and CN, with some evidence of lower v_*p*_ in the CP group (Supplementary Tables 4, 5 and Figure 4). Collectively these data demonstrate BBB disruption in PD can be detected in the clinical setting in keeping with evidence from studies in animal models and post mortem human brain. The *K*^trans^ values (Figure 4) are within the wide range of published values which seem dependent on the specific acquisition and analysis methods and contrast agents used (Raja et al., 2018). A study using the same contrast agent and similar method shows very comparable values (Heye et al., 2016).

Both the voxel-based and the ROI analysis showed higher *K*^trans^ in PD when compared with CN. These results are in keeping with several studies showing altered components of the BBB in PD (Barcia et al., 2005; Carvey et al., 2005; Chao et al., 2009; Sarkar et al., 2014) such as loss of capillaries, an alteration in the capillary calibre and thickened basement membrane (making the BBB less competent) (Brown and Thore, 2011). Our voxel-based analysis approach allows a whole brain view of BBB dysfunction, and, in the whole brain maps, we see a fairly diffuse pattern of BBB disruption in PD, compared to CN. *K*^trans^ differences only reach statistical significance in posterior regions; however, given the requirement for multiple comparisons correction, it would likely require a much larger study for smaller brain regions such as basal ganglia nuclei to survive the statistical threshold. The ROI approach focussed on areas expected to display disease pathology based on our understanding of the pathophysiology of PD. It revealed *K*^trans^ to be generally higher in the PD group than in the CN group. Considering the magnitude and significance of regional *K*^trans^ differences between PD and CN (Figure 4), this is driven mainly by differences in SN, NAWM, WML and the posterior cortex. Alterations in SN and posterior cortex are in keeping with BBB breakdown playing a role in the pathophysiology of PD.

The increased *K*^trans^ in posterior cortical regions in PD is particularly noteworthy as these are the same regions that display hypoperfusion (Borghammer et al., 2010; Melzer et al., 2011; Fernandez-Seara et al., 2012; Al-Bachari et al., 2017) i.e., the results strengthen the argument of a link between BBB leakage and hypoperfusion. Hypoperfusion has been attributed to altered vasculature (string vessels, shorter/loss of capillaries, tortuous vessels), which can hinder normal BBB function (Rite et al., 2007; Guan et al., 2013; Gray and Woulfe, 2015; Fang, 2018). BBB disruption has been attributed to alterations in key components such as tight junctions, potentially caused by pro-inflammatory cytokines and altered vascular endothelial growth factor (VEGF) (Carvey et al., 2009; Matsumoto et al., 2017; Chen et al., 2018). Interestingly a study using albumin (mg/L)/plasma albumin (g/L) ratio in the cerebral spinal fluid (CSF) to measure BBB dysfunction, revealed increased BBB dysfunction in PD compared to controls which was associated with increased CSF biomarkers of angiogenesis (e.g., VEGF) (Janelidze et al., 2015). These substances can also alter perfusion, with enhanced angiogenesis resulting in abnormal vascular permeability in PD. Future longitudinal imaging studies will be important to understand whether hypoperfusion leads to altered BBB function, or vice versa, or in fact whether BBB and perfusion changes have a common cause, for example, inflammation. Furthermore, it will be important to determine what downstream effects these microvascular changes may have on neuronal loss. We did not observe any statistically significant differences in *K*^trans^ between CP and CN unlike other studies which have reported elevated *K*^trans^ post-stroke (Wardlaw et al., 2017). However, the small sample size and high heterogeneity of the CP group may have contributed to this.

White matter lesion have been used as a surrogate marker of SVD (Wardlaw et al., 2013). We find higher WML volume in the PD group than the CN group despite the fact the groups have no significant differences in cardiovascular risk factors. Previous studies of WML in PD however show mixed results (Rabbitt et al., 2006; de Schipper et al., 2019). To investigate whether the WML volume was driving the *K*^trans^ group differences, we used ANOVA with WML volume, age, and gender as covariates and found that there was no significant association between WML volume (or age or gender) and *K*^trans^. The main finding of higher *K*^trans^ in the PD group than CN was maintained. Recent work has tried to understand the link between BBB dysfunction and WML revealing a continuum of BBB disruption leading to myelin loss and fibrinogen accumulation resulting in WML formation (Muñoz Maniega et al., 2017; Wardlaw and William, 2018; Wardlaw et al., 2019). Indeed NAWM (particularly that surrounding the WML) has been shown to have increased BBB leakage suggesting BBB disruption can precede WML formation (Wardlaw et al., 2017). We do not find a significant association between WML volume and *K*^trans^, which alongside the higher *K*^trans^ in NAWM in the PD group compared to CN, supports this notion that BBB disruption is diffuse throughout the white matter and perhaps precedes WML formation. It is interesting to note that, within the lesions, *K*^trans^ is significantly higher in the PD group than in the CN group suggesting more severe underlying pathology.

Our measurements of blood plasma volume, v_*p*_ are not significantly different between PD and healthy controls, which may seem contradictory to the well-reported hypoperfusion in PD, given that blood volume and perfusion are closely related. However, blood flow also depends on the blood velocity within the capillaries which may underlie the observed differences. Indeed, we have previously found significantly prolonged arterial transit time in posterior brain regions in PD, suggesting lower blood velocity (Al-Bachari et al., 2017). Furthermore, v_*p*_ estimation may also be affected by the rate of *trans*-endothelial water exchange leading to possible underestimation of v_*p*_ in the CN group in comparison to the PD group due to the relatively more intact BBB, potentially contributing to the lack of group difference seen. As the *K*^trans^ and v_*p*_ patterns differed between CP and PD this would suggest that the BBB alterations do not simply occur due to co-existing cerebrovascular disease (indeed PD patients with known cerebrovascular disease were not included in the study and the PD group had significantly fewer cerebrovascular risk factors) but plays an independent role in PD pathophysiology. Together with the increased WML volume in the PD group, this supports the hypothesis that microvascular pathology occurs in PD.

We explored the impact of cognitive deficit (MoCA score), medication (LEDD dose) and disease severity (UPDRS score) on *K*^trans^ and v_*p*_ within the PD group. LEDD dose was associated with higher v_*p*_, which is in keeping with studies that suggest L-dopa increases blood flow in certain regions (Ohlin et al., 2012; Ko et al., 2015). There were no significant associations between any parameter and *K*^trans^ and we conclude that the *K*^trans^ differences between the PD and CN groups are not driven by the differences in MoCA score or by levodopa medications.

One limitation of this study is the significant gender imbalance between the PD and CN groups (Table 1) with relatively more men in the PD group. However, we do not believe this compromises our findings as there are no reports of gender differences in *K*^trans^ values, and secondary analysis of our own data shows consistent findings in a gender-matched sub-group (Supplementary Materials). Likewise we collected data on two different scanners which may have influenced the results; however secondary analysis shows consistent findings in analysis of data from a single scanner (Supplementary Materials). We interpret the higher *K*^trans^ in PD as relating to higher endothelial leakage, but note that *K*^trans^ is also affected by cerebral blood flow. However cerebral blood flow is lower than normal in posterior regions in PD (Barcia et al., 2005; Kortekaas et al., 2005; Tomlinson et al., 2010; Gray and Woulfe, 2015) which would lead to lower *K*^trans^, and yet we see higher *K*^trans^ in the PD group, implying that the differences are not due to blood flow differences.

## Conclusion

In conclusion, this study has shown subtle BBB disruption in PD, in key regions implicated in the pathophysiology including the SN, white matter and posterior cortical regions. Further research is needed, including longitudinal clinical imaging studies combining neuronal, metabolic and vascular measurements to better understand disease mechanisms and so identify potential therapeutic targets in PD.

## Data Availability Statement

Data including images, imaging metrics and participant metadata are available on request. Please email the corresponding author: Laura.Parkes@manchester.ac.uk.

## Ethics Statement

The studies involving human participants were reviewed and approved by NHS ethical approval (North West – Preston Research Ethics Committee), United Kingdom. The patients/participants provided their written informed consent to participate in this study.

## Author Contributions

SA-B contributed to the conception of the study, organised and executed the study, collected all data, performed the majority of the data analysis and interpretation, wrote the first draft of the manuscript, and contributed to further editing. JN and GP contributed to the execution of the study and the data analysis and interpretation as well as reviewing the manuscript. HE conceived of and organised the study, contributed to data interpretation, and reviewed and edited the manuscript. LP contributed to the conception, organisation and execution of the study, contributed to the data analysis and interpretation, and reviewed and edited the manuscript. All authors contributed to the article and approved the submitted version.

## Conflict of Interest

Authors JN and GP were part employed by the company Bioxydyn and hold shares in the company. The remaining authors declare that the research was conducted in the absence of any commercial or financial relationships that could be construed as a potential conflict of interest.

## Footnotes

1 https://www.ncbi.nlm.nih.gov/projects/gap/cgi-bin/GetPdf.cgi?id=phd000042

2 https://www.movementdisorders.org/MDS/MDS-Rating-Scales/MDS-Unified-Parkinsons-Disease-Rating-Scale-MDS-UPDRS.htm

3 www.MoCAtest.org

4 https://www.statistical-modelling.de/lst.html

5 www.fil.ion.ucl.ac.uk/spm

6 https://www.alivelearn.net/xjview/

7 https://rstudio.com/products/rstudio/

## Abbreviations

ANOVA: analysis of variance
BBB: blood–brain barrier
CA: caudate
CN: control negative
CP: control positive
CSF: cerebral spinal fluid
DCE-MRI: dynamic contrast enhanced magnetic resonance imaging
FLAIR: fluid attenuation inversion recovery
FC: frontal cortex
FWE: family wise error
Hct: haematocrit
*K*^trans^: contrast agent endothelial transfer coefficient
LEDD: levodopa equivalent daily dose
MNI: Montreal Neurological Institute
MoCA: Montreal cognitive assessment
NAWM: normal appearing white matter
NHS: National Health Service
P: pallidum
PET: positron emission tomography
PC: posterior cortices
PD: Parkinson’s disease
PU: putamen
ROI: region of interest
SN: substantia nigra
SNpc: substantia nigra pars compacta
SPM: statistical parametric mapping
T_1_-FFE: T_1_ fast field echo
TE: echo time
3 T: 3 tesla
TR: repetition time
UPDRS: Unified Parkinson’s Disease Rating Scale
v_*p*_: plasma volume
VEGF: vascular endothelial growth factor
WML: white matter lesion.
