# Supplementary material for: Blood–Brain Barrier Leakage Is Increased in Parkinson’s Disease
Source: Front Physiol. 2020 Dec 22;11:593026. doi: 10.3389/fphys.2020.593026 (PMC7784911; doi:10.3389/fphys.2020.593026)
Supplement: Supplementary file 1 [file Data_Sheet_1.pdf]

Supplementary Material

1. Methods

Further detail of MRI acquisition protocol and analysis is outlined below.

1.1 MRI acquisition protocol

A DCE-MRI dynamic series of 3D T_1-fast field echo (T_1-FFE) were acquired with the following scan parameters: Field of view 192 mm x 192 mm and matrix size of 128 giving in-plane resolution of 1.5 x 1.5 mm; 32 contiguous axial slices of 4 mm thickness; echo time (TE) = 0.8 ms, repetition time (TR) = 2.4 ms, flip angle 10 degrees and image acquisition time of 7.6 seconds. 160 images were acquired over approximately 20 minutes.

Prior to the dynamic scan, a series of additional 3D T_1-FFE images were acquired at 3 flip angles (2, 5 and 10 degrees) in order to calculate a pre-contrast T_1 map using the variable flip angle method (1), with geometry and all other parameters matched to the dynamic series, except only 8 image repeats were collected (from which a mean image was created), giving an acquisition time of 60 s per flip angle. In order to correct for B_1 field inhomogeneities, a B_1 mapping sequence (2) was also acquired with the same voxel size and coverage as for the variable flip angle images. This consisted of a pair of 3D spoiled gradient echo images with TR_1 = 25 ms and TR_2 = 125 ms, flip angle 60 degrees, TE = 5 ms, acquisition time 117 seconds.

In addition, a T_2-weighted FLAIR image was acquired with the following parameters: TR = 10 s, inversion time 2.75 s, TE = 140 ms, in-plane resolution of 0.69 mm x 0.69 mm, and 100 contiguous axial slices of 1.3 mm thickness with an acquisition time of 450 seconds. A 3D T_1-weighted image was also collected with scan parameters: TR = 8.4 ms, TE = 3.9 ms, flip angle 8 degrees. Images were reconstructed with a resolution of 0.94 mm x 0.94 mm x 1mm, acquisition time 311 seconds.

1.2 MRI analysis

A voxel-by-voxel fit of the dynamic data for both the contrast agent transfer coefficient (K^{trans}) and plasma volume (v_p) was performed using the 'Patlak' model:

$$C_t(t) = K^{trans} \int_0^t C_p(t')dt' + v_p C_p(t) \qquad [1]$$

Where $C_t(t)$ is the tissue concentration of the contrast agent and $C_p(t)$ is the plasma concentration at time t after contrast agent injection at t=0.

The tissue concentration $C_t(t)$ is calculated from the signal in the dynamic images $S_t(t)$ according to:

$$C_{t,b}(t) = \frac{R_1(t) - R_{10}}{r_1} \qquad [2]$$

Where $R_1(t)$ is calculated from $S_t(t)$ as described subsequently in equation 5. Where r_1 is the longitudinal relativity of the contrast agent, which was assumed to be 3.4 $s^{-1}mM^{-1}$. R_{10} is the baseline longitudinal relaxation rate, taken from the pre-contrast T_1 map using $R_{10} = 1/T_{10}$.

This pre-contrast T_1 map was calculated by fitting the variable flip angle images on a voxel-by-voxel basis for T_{10} and A_0 using equation [3] (1):

$$S = \frac{A_0 \sin\theta (1-e^{-TR/T_{10}})}{1-\cos\theta e^{-TR/T_{10}}} \qquad [3]$$

In order to correct for inaccuracies in the specified flip angles, θ_S, due to B_1 inhomogeneities, the ratio of the image intensities in the B_1 mapping sequence (r) was used to estimate the true flip angle θ_T on a voxel-by-voxel basis (3) , using:

$$\theta_T = \cos^{-1}\left(\frac{r.n-1}{n-r}\right) \qquad \text{where } n=TR_1/TR_2. \qquad [4]$$

The deviation of the true flip angle from the specified flip angle (θ_S) is given by θ_T/θ_S and θ in equation [3] is multiplied by this factor on a voxel wise basis when calculating T_1. Prior to multiplication, the θ_T/θ_S image was smoothed using a convolution kernel of 3 voxels.

$R_1(t)$ was calculated using equation [5] below, derived from equation [3], considering S_t as the signal in the post-contrast dynamic images and S_0 as the mean signal from 5 pre-contrast dynamics, ignoring the first image due to equilibrium effects.

$$R_1(t) = -\frac{1}{TR}\ln\left[\frac{1-B\cos\theta+\frac{S_t(t)}{S_0}(B-1)}{1-B\cos\theta+\frac{S_t(t)}{S_0}\cos\theta(B-1)}\right] \qquad \text{where } B=\exp(-R_{10}.TR). \qquad [5]$$

Finally, the plasma concentration $C_p(t)$, is derived from the blood concentration $C_b(t)$ which is also calculated using equations [2] and [5] with S_t the mean signal from the sagittal sinus region, S_0 the mean signal from 5 pre-contrast dynamics within this region (again, neglecting the first one to avoid inflow effects), and T_{10} equal to the mean pre-contrast T_1 from the sagittal sinus region. $C_p(t)$ is derived from the measured blood concentration by correcting for hematocrit according to: $C_p(t) = C_b(t)/(1\text{-Hct})$ where Hct of 0.40 was used for a female and 0.45 for male (4).

Equation [1] was then fit to $C_t(t)$ and $C_p(t)$ using constrained least squares minimisation (lsqcurvefit in Matlab) on a voxel-wise basis for 3 parameters: K^{trans}, v_p and T_0, where T_0 is the offset time between $C_t(t)$ and $C_p(t)$. K^{trans} was constrained to be between -0.001 min^{-1} and 0.1 min^{-1}, v_p between 0 and (1-Hct) and T_0 between -20 and 20 s The non-zero lower bound on K^{trans}, while un-physiological, is to avoid positive bias in the K^{trans} values which may in reality be very close to zero in healthy tissue. The negative bounds on T_0 are to allow for the possibility that the peak sagittal sinus signal may occur earlier than that of the regional blood circulation if blood takes a particularly tortuous path to the region. In order to avoid local minima, minimisation was performed twice, using the fitted parameters of the first minimisation as starting parameters for the second minimisation, except for one parameter which was kept as the original value.

Voxel-wise analysis was performed using the SPM12 PET toolbox to determine regional differences in K^{trans} and v_p between the groups. First we co-registered the K^{trans} and v_p maps to the high resolution 3D T_1-weighted image: the first volume of the motion-corrected DCE series was used to compute the registration parameters which were then applied to the other images. Each T_1-weighted image was segmented into tissue classes using SPM and grey and white matter masks were defined using a probability threshold of 75%. The co-registered

parameter maps were masked to include signal only from grey and white matter. The T_1-weighted images were then normalized to Montreal Neurological Institute (MNI) space and the transformation applied to the K^{trans} and v_p maps.

1.3 Visual inspection of data quality

The DCE concentration time course in the white matter and the sagittal sinus regions (i.e. the VIF) were extracted for each person in order to consider individual differences in this raw data prior to fitting. For example, attention was paid to the shape and amplitude of the VIF in order to ascertain that the contrast agent injection was as expected.

Figure S1 shows example tissue concentration time courses for a representative person from each group, selected according to their K^{trans} and v_p values being close to the median for the group. It can be seen that, while the vascular input functions are very similar (Figure S1A), the tissue curves begin to diverge, particularly for the PD group (Figure S1B). Baseline T_1 values did not vary significantly between the groups, with mean ± SE values of CN: 1.70 s ± 0.04 s, CP: 1.76 s ± 0.04 s, PD: 1.74 s ± 0.04 s.

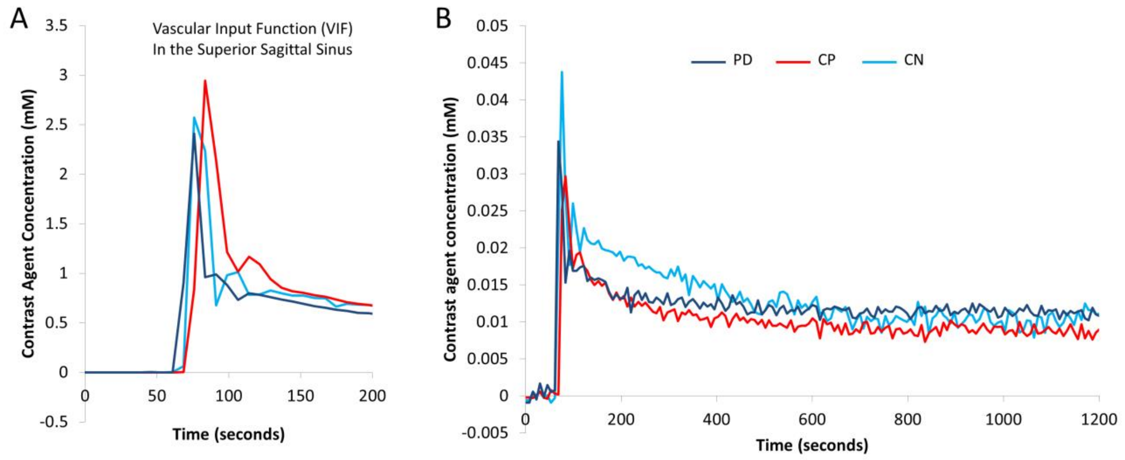

Figure S1: Concentration time-course of contrast agent in blood and tissue
Typical time-courses of the vascular input function (A) and the white matter contrast agent concentration (B) taken from representative subjects of each group. Subjects were selected according to K^{trans} and v_p values being close to the median for the group.

2. Results

2.1 Voxel-wise analysis

Tables S1, S2,S3, S4 and S5 provide details of the regions showing group differences in the voxel-wise analysis, highlighting regions of significant difference in K^{trans} between the PD and CN groups (Table S1), the PD and CP groups (Table S2) and the CP and CN groups (Table S3) and regional differences in v_p between the CP and CN groups (Table S4) and CP and PD groups (Table S5).

Table S1. Regions of significantly higher K^{trans} in the PD group than in the CN group. The t-values are thresholded with voxel-level p < 0.001 (uncorrected) and minimum cluster size of 50 voxels.

Region	Cluster size	Cluster p (FWE corr)	Peak t-value	Peak p (uncorr.)	Peak MNI coordinates
White matter in the R hemisphere precuneus extending into parietal lobe and calcarine	406	0.04	3.96<br>3.87<br>3.48<br>3.36<br>3.35	<0.0001<br>0.0001<br>0.0004<br>0.0006<br>0.0006	22 -56 32<br>32 -40 22<br>28 -36 28<br>16 -50 10<br>12 -32 30
White matter in the L and R precuneus extending into paracentral lobule	306	0.1	3.86<br>3.73<br>3.60<br>3.54<br>3.38	0.0001<br>0.0002<br>0.0003<br>0.0003<br>0.0006	-6 -40 64<br>12 -36 60<br>8 -36 66<br>-12 -28 52<br>-12 -38 60
R inf temporal gyrus	82	0.7	3.7	0.0001	50 -26 -26

Table S2. Regions of significantly higher K^{trans} in the PD group than in the CP group. The t-values are thresholded with voxel-level p < 0.001 (uncorrected) and minimum cluster size of 50 voxels.

Region	Cluster size	Cluster p (FWE corr)	Peak t-value	Peak p (uncorr.)	Peak MNI coordinates
White matter in R temporal lobe	69	0.8	3.73	0.0002	34 -60 16

Table S3. Regions of significantly higher K^{trans} in the CP group than in the CN group. The t-values are thresholded with voxel-level p < 0.001 (uncorrected) and minimum cluster size of 50 voxels.

Region	Cluster size	Cluster p (FWE corr)	Peak t-value	Peak p (uncorr.)	Peak MNI coordinates
R cerebellum	233	0.1	3.96<br>3.85	0.0001<br>0.0002	34 -80 -30<br>20 -82 -24
Midline cingulate gyrus	104	0.6	3.99<br>3.79	0.0001<br>0.0002	0 -24 28<br>0 -16 32

Table S4. Regions of significantly lower v_p in the CP group than in the CN group. The t-values are thresholded with voxel-level p < 0.001 (uncorrected) and minimum cluster size of 50 voxels.

Region	Cluster size	Cluster p (FWE corr)	Peak t-value	Peak p (uncorr.)	Peak MNI coordinates
White matter of L temporal lobe	242	0.3	4.46<br>4.04<br>3.76	<0.0001<br>0.0001<br>0.0003	-36 -40 4<br>-32 -54 6<br>-22 -68 8
White matter of R temporal lobe extending into insula and sup. temporal gyrus	290	0.2	4.34<br>4.16<br>4.12<br>3.92	<0.0001<br><0.0001<br><0.0001<br>0.0002	38 -34 0<br>40 -22 -4<br>38 -26 -2<br>42 -22 4
L sup. temporal gyrus	53	0.8	3.80	0.0002	-40 -60 26

Table S5. Regions of significantly lower v_p in the CP group than in the PD group. The t-values are thresholded with voxel-level p < 0.001 (uncorrected) and minimum cluster size of 50 voxels.

Region	Cluster size	Cluster p (FWE corr)	Peak t-value	Peak p (uncorr.)	Peak MNI coordinates
White matter of the R temporal lobe	65	0.8	4.00	<0.0001	40 -34 -4
White matter of the L temporal lobe	85	0.7	3.93<br>3.67	0.0001<br>0.0003	-36 -40 0<br>-34 -46 4

2.2 ROI analysis with scanner and gender matching

To check that any apparent group differences in imaging metrics were not driven by group differences in gender or scanner used, we repeated the ROI analysis on a selected group of participants with these factors matched across groups.

2.21 ROI analysis with scanner matching

Table S6 demonstrates the demographics for participants whose data was collected on the same scanner at the Manchester Clinical Research Facility. Figure S2 shows regional values for K^{trans} and v_p using data collected from these groups. The results are in broad agreement with the results reported in the main paper (Figure 4). ANOVA with K^{trans} as the dependent variable and WML volume, age and gender included as covariates showed a significant effect of group (F = 3.5, p=0.04) and region (F = 44.6, p<0.0001) on K^{trans} and no significant effect of WML volume (F=0.4, p=0.5), age (F=0.4, p=0.5) or gender (F=0.001, p=1.0). Post-hoc tests again showed K^{trans} to be significantly higher in PD than in CN (p=0.03, Bonferroni corrected) and no significant differences between the other two pairwise comparisons. Similar analysis with v_p as the dependent variable showed a significant effect of region (F =

55.2, p<0.0001) but not group (F = 0.9, p=0.4) on v_p and no significant effect of WML volume (F=0.02, p=0.9), age (F=1.6, p=0.2) or gender (F=0.05, p=0.8).

Table S6: Demographics and clinical and radiological characteristics of the participants for data collected only on the same scanner

	**CN (n=20)**	**CP (n=15)**	**PD (n=36)**	**p value** PD v CN	**p value** PD v CP	**p value** CP v CN
**n** (F:M)	13:7	4:11	12:37	0.002	0.26	0.02
**Age (years):** mean (range)	67.8 (51-81)	69.1 (53-84)	70 (52-85)	0.31	0.69	0.64
**No. of cardiovascular risk factors:** mean (SD)	1.71 (1.31)	2.93 (1.16)	1.72 (1.52)	0.92	0.009	0.006
**Cardiovascular Risk Factors (% of group):**<br>Hypertension<br>Diabetes mellitus<br>FH of CVD<br>Smoker<br>Hypercholesterolaemia<br>Ischaemic heart disease<br>Atrial fibrillation	 <br>35.0<br>5.0<br>50.0<br>25.0<br>45.0<br>10.0<br>0	 <br>73.3<br>13.3<br>46.7<br>66.7<br>68.9<br>13.3<br>20.0	 <br>38.9<br>8.3<br>36.1<br>38.9<br>36.1<br>16.7<br>0	 <br>0.22<br>0.39<br>0.13<br>0.14<br>0.18<br>0.26<br>1	 <br>0.02<br>0.43<br>0.19<br>0.05<br>0.01<br>0.32<br>0.02	 <br>0.02<br>0.50<br>0.26<br>0.01<br>0.07<br>0.38<br>0.07
**Disease Duration (years):** mean (SD)	N/A	1.1 (0.77)	6.9 (4.38)	N/A	N/A	N/A
**Hoehn & Yahr Score:** mean (SD)	N/A	N/A	2.61 (0.95)	N/A	N/A	N/A
**UPDRS Score:** mean (SD)	N/A	N/A	30.4 (11.6)	N/A	N/A	N/A
**LEDD (mg):** mean (SD)	N/A	N/A	577.5 (329.2)	N/A	N/A	N/A
**MoCA Score:** mean (SD)	27.7 (2.0)	26.1 (2.9)	24.9 (4.1)	0.001	0.22	0.08
**Cube-root of WML volume (mm):** mean (SD)	**1.37 (0.93)**	**2.11 (0.72)**	**2.08 (0.91)**	**0.009**	0.89	**0.01**

CN: control negative; CP: control positive; FH of CVD: family history of cardiovascular disease; LEDD: levodopa equivalent daily dose; MoCA: Montreal Cognitive Assessment; PD: Parkinson's disease; UPDRS 111: unified Parkinson's disease rating scale motor score; WML: white matter lesion.

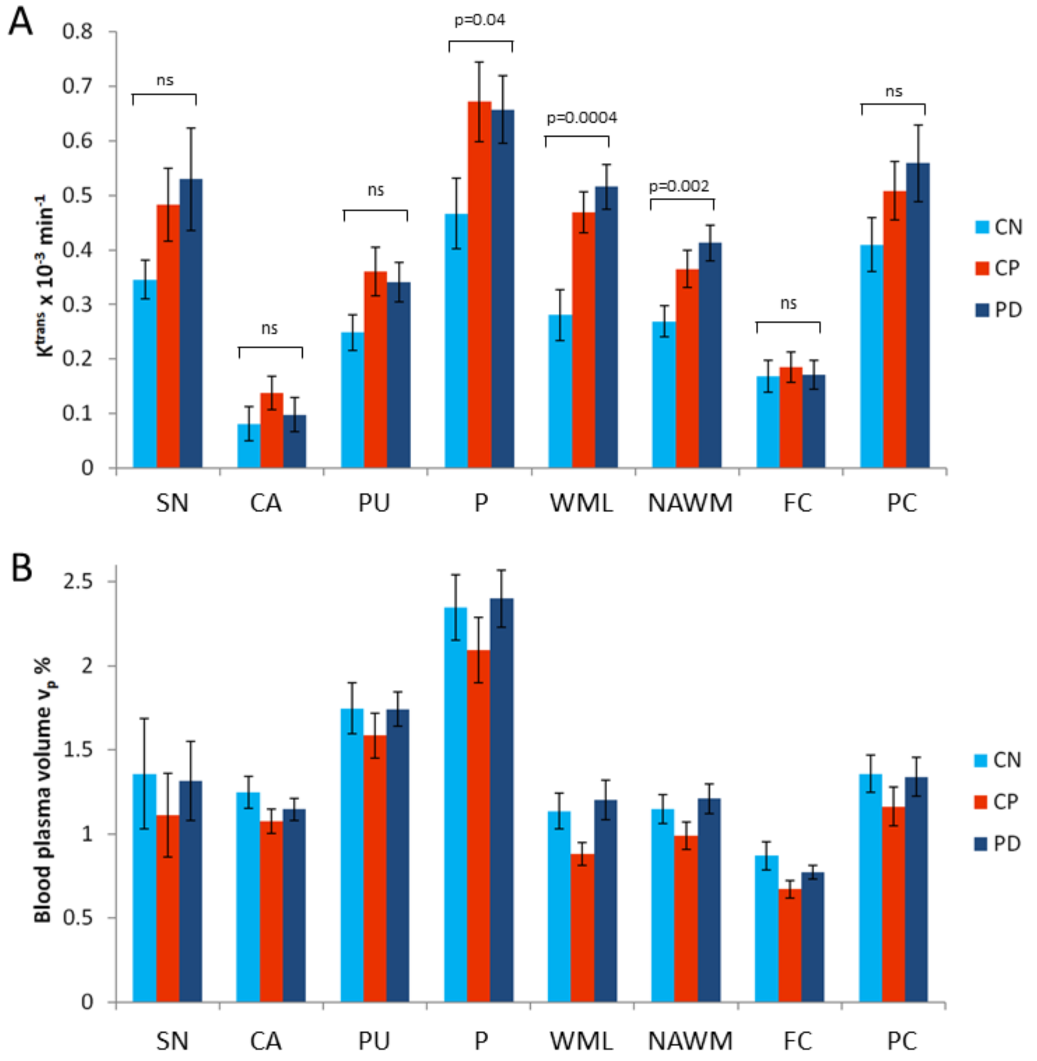

Figure S2: Mean group values for K^{trans} and v_p in regions of interest from data collected on the same scanner

Mean values are given for the (A) the contrast agent transfer coefficient K^{trans} and (B) the plasma volume v_p. Error bars show the standard error in the mean. SN = substantia nigra, CA= caudate, PU = putamen, P = pallidum, WML = white matter lesions, NAWM = normal-appearing white matter, FC = frontal cortex, PC = posterior cortices.

2.22 ROI analysis with gender matching

Table S7 shows the demographics and clinical characteristics of the gender matched groups. Figure S3 show regional values for K^{trans} and v_p using data from these groups. The results are in broad agreement with the results reported in the main paper (Figure 4). ANOVA with K^{trans} as the dependent variable and WML volume, age and gender included as covariates showed a significant effect of group ($F = 3.0$, $p=0.05$) and region ($F = 51.1$, $p<0.0001$) on K^{trans} and no significant effect of WML volume ($F=0.04$, $p=0.8$), age ($F=2.2$, $p=0.14$) or gender ($F=0.1$, $p=0.7$). Post-hoc tests again showed K^{trans} to be significantly higher in PD than in CN ($p=0.04$, Bonferroni corrected) and no significant differences between the other two pairwise comparisons. Similar analysis with v_p as the dependent variable showed a significant effect of region ($F = 88.1$, $p<0.0001$) but not group ($F = 0.8$, $p=0.5$) on v_p and no significant effect of WML volume ($F=0.01$, $p=0.9$), age ($F=1.0$, $p=0.3$) or gender ($F=0.5$, $p=0.5$).

Table S7: Demographics and clinical and radiological characteristics of the gender matched study group

	**CN (n=21)**	**CP (n=15)**	**PD (n=49)**	**p value** PD v CN	**p value** PD v CP	**p value** CP v CN
**n** (F:M)	6:15	4:11	12:37	0.20	0.25	0.29
**Age (years):** mean (range)	67.1 (52-81)	69.1 (53-84)	68.9 (52-85)	0.42	0.84	0.44
**No. of cardiovascular risk factors:** mean (SD)	1.71 (1.31)	2.93 (1.16)	1.72 (1.52)	0.97	0.002	0.003
**Cardiovascular Risk Factors (% of group):**						
Hypertension	33.3	73.3	26.5	0.20	0.02	0.02
Diabetes mellitus	9.5	13.3	6.1	0.32	0.43	0.44
FH of CVD	52.4	46.7	22.4	0.06	0.15	0.25
Smoker	23.8	66.7	28.6	0.13	0.03	0.01
Hypercholesterolaemia	38.1	68.9	22.4	0.13	0.004	0.05
Ischaemic heart disease	9.52	13.3	12.2	0.24	0.31	0.37
Atrial fibrillation	0	20.0	2.0	0.7	0.04	0.06
**Disease Duration (years):** mean (SD)	N/A	1.1 (0.77)	7.2 (4.45)	N/A	N/A	N/A
**Hoehn & Yahr Score:** mean (SD)	N/A	N/A	2.60 (0.09)	N/A	N/A	N/A
**UPDRS Score:** mean (SD)	N/A	N/A	29.2 (12.7)	N/A	N/A	N/A
**LEDD (mg):** mean (SD)	N/A	N/A	583.5 (399.6)	N/A	N/A	N/A
**MoCA Score:** mean (SD)	27.7 (2.4)	26.1 (2.9)	25.3 (3.9)	0.004	0.39	0.10
**Cube-root of WML volume (mm):** mean (SD)	**1.26 (0.81)**	**2.11 (0.72)**	**1.80 (0.95)**	**0.02**	0.19	**0.002**

CN: control negative; CP: control positive; FH of CVD: family history of cardiovascular disease; LEDD: levodopa equivalent daily dose; MoCA: Montreal Cognitive Assessment; PD: Parkinson's disease; UPDRS 111: unified Parkinson's disease rating scale motor score; WML: white matter lesion

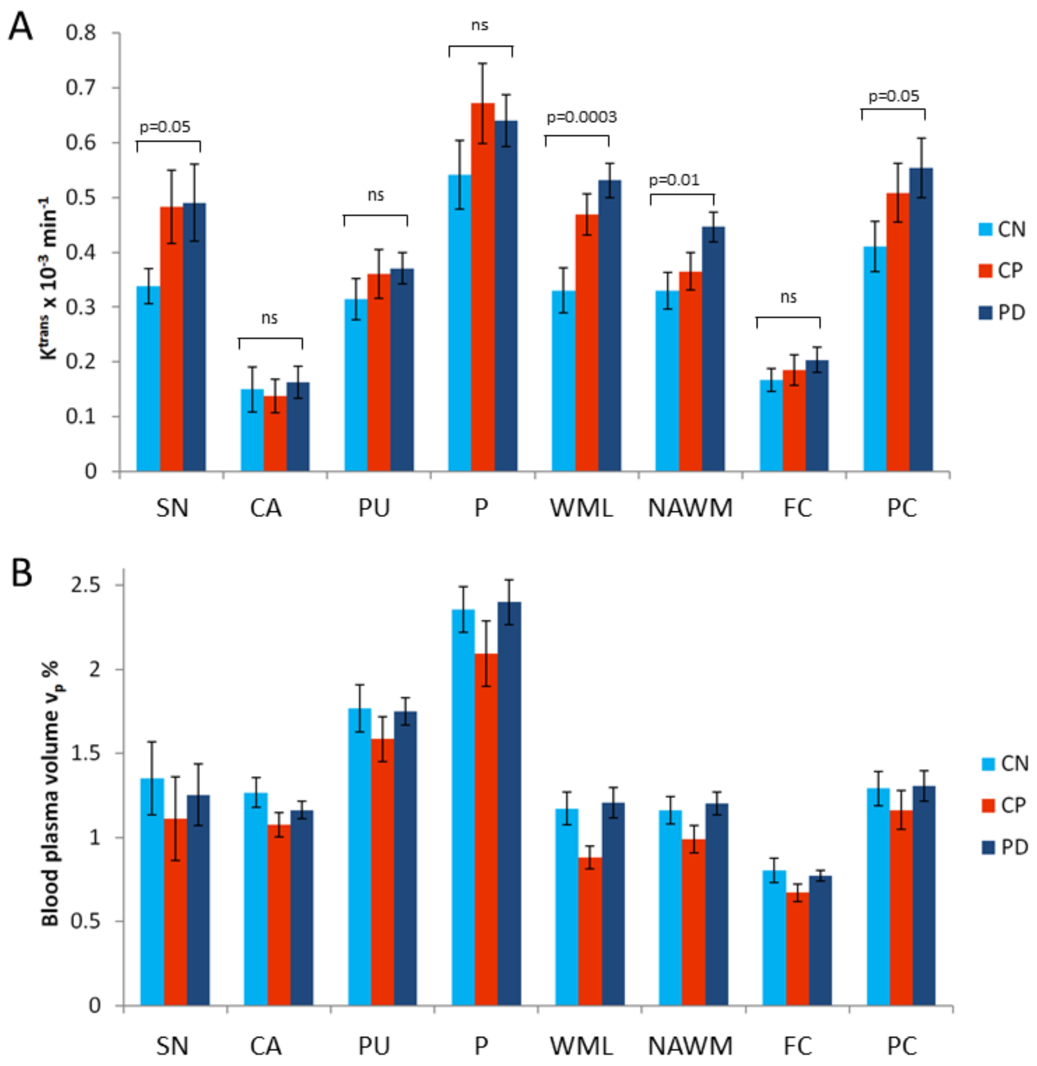

Figure S3: Mean group values for K^{trans} and v_p in regions of interest with matched gender balance between groups

Mean values are given for the (A) the contrast agent transfer coefficient K^{trans} and (B) the plasma volume v_p. Error bars show the standard error in the mean. SN = substantia nigra, CA= caudate, PU = putamen, P = pallidum, WML = white matter lesions, NAWM = normal-appearing white matter, FC = frontal cortex, PC = posterior cortices.

References

1. Fram EK, Herfkens RJ, Johnson GA, Glover GH, Karis JP, Shimakawa A, et al. Rapid calculation of T1 using variable flip angle gradient refocused imaging. Magn Reson Imaging. 1987;5(3):201-8.
2. Voigt T, Nehrke K, Doessel O, Katscher U. T1 corrected B1 mapping using multi-TR gradient echo sequences. Magn Reson Med. 2010;64(3):725-33.
3. Pohmann R, Scheffler K. A theoretical and experimental comparison of different techniques for B(1) mapping at very high fields. NMR Biomed. 2013;26(3):265-75.
4. Cirillo M, Laurenzi M, Trevisan M, Stamler J. Hematocrit, blood pressure, and hypertension. The Gubbio Population Study. Hypertension. 1992;20(3):319-26.
